# Rare triad of anomalous biliary anatomy (pancreaticobiliary maljunction), choledochal cyst and cholangiocarcinoma in a 45-year-old white male: A case report

**DOI:** 10.1016/j.radcr.2024.10.032

**Published:** 2024-10-26

**Authors:** Brian Markovich, Cara Lombard, Brian A. Boone, Shyam Thakkar, Nicholas A. Puleo, Subtain Ali

**Affiliations:** a Department of Radiology, West Virginia University, Morgantown, WV, USA; b Department of Surgery, West Virginia University, Morgantown, WV, USA; c Department of Medicine - Division of Gastroenterology & Hepatology, West Virginia University, Morgantown, WV, USA

**Keywords:** Anomalous biliary anatomy, Choledochal cyst, Cholangiocarcinoma, Pancreaticobiliary maljunction

## Abstract

Pancreaticobiliary maljunction (PBM) is a congenital anomaly where the pancreatic and bile ducts join outside the duodenal wall, resulting in formation of an elongated common channel. In normal physiology, the sphincter of Oddi regulates the junction between the pancreatic and bile ducts. Individuals with PBM lack this regulatory mechanism resulting in reflux of pancreatic juices into the biliary tract. Activated pancreatic enzymes result in chronic inflammation of biliary tract resulting in choledochal cysts which places patients at risk for eventual development of cholangiocarcinoma.

A 45-year-old white male presented with jaundice and dark urine. Laboratory tests showed elevated liver enzymes and bilirubin. Diagnostic imaging revealed anomalous biliary anatomy, a Type 1A choledochal cyst, and a mass in the common hepatic duct. Extrahepatic cholangiocarcinoma was confirmed by ERCP and biopsy, with PET/CT indicating localized disease without distant metastases.

Treatment included a biliary sphincterotomy, stone drainage, and stenting. The patient underwent a robotic-assisted bile duct resection, cholecystectomy, hepatic lobectomy, and Roux-en-Y hepaticojejunostomy. The surgical specimen showed an invasive, poorly differentiated adenosquamous carcinoma with perineural invasion, but no regional lymph node involvement.

PBM is a rare condition, and its diagnosis and management necessitate a multidisciplinary team, including pancreaticobiliary surgeons, endoscopists, and radiologists. Accurate diagnosis hinges on the team's expertise. Radiologists must be aware of PBM and have a thorough understanding of the associated anatomy and imaging characteristics that may indicate high-risk dysplasia or malignancy.

## Introduction

This case highlights a rare triad of pancreaticobiliary maljunction, Type 1A choledochal cyst, and cholangiocarcinoma in a 45-year-old male. The diagnosis was confirmed using a combination of advanced imaging techniques and endoscopic evaluation. This article examines the diagnostic workup, treatment options, pathology, and clinical recommendations related to this presentation.

## Case presentation

A 45-year-old white male who presented to the emergency department with a 2-day history of jaundice and dark-colored urine. Otherwise, he reported feeling generally well with no specific complaints. He denied abdominal pain, nausea, vomiting, recent illness, alcohol abuse, or illicit drug use. Furthermore, there was no history of hepatitis or chronic liver disease. Laboratory evaluation was notable for elevated levels of alkaline phosphatase (546 U/L), AST (174 U/L), and bilirubin (8.7 mg/dL).

**Fig. 1:**
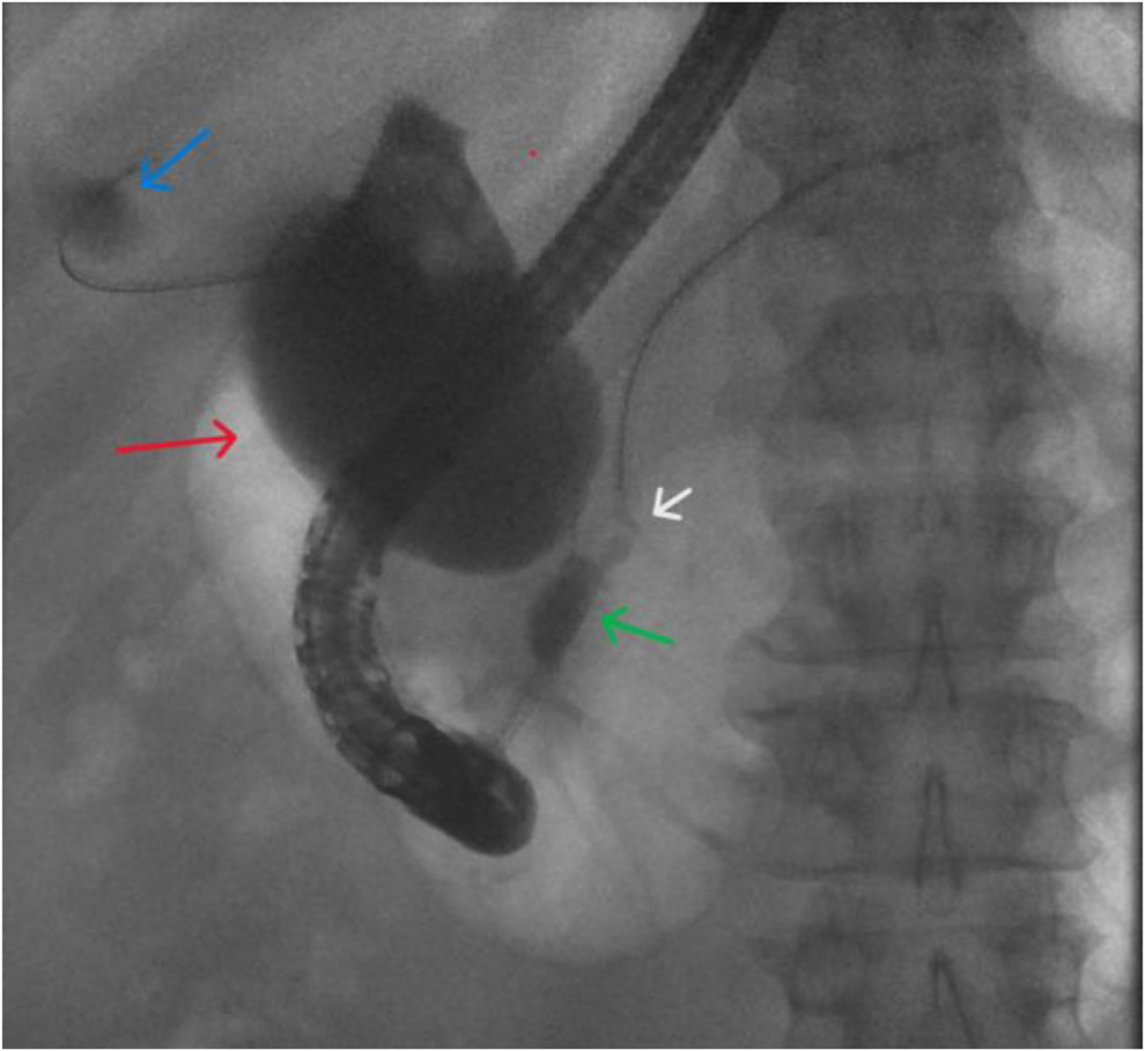
Intraoperative endoscopic retrograde cholangiogram demonstrates a long common channel (green arrow) joining the pancreatic duct (white arrow) with the common bile duct. Note the fusiform dilation of the common bile duct (red arrow) into which the gallbladder (blue arrow) and cystic duct drain consistent with Type 1A choledochal cyst.

**Fig. 2:**
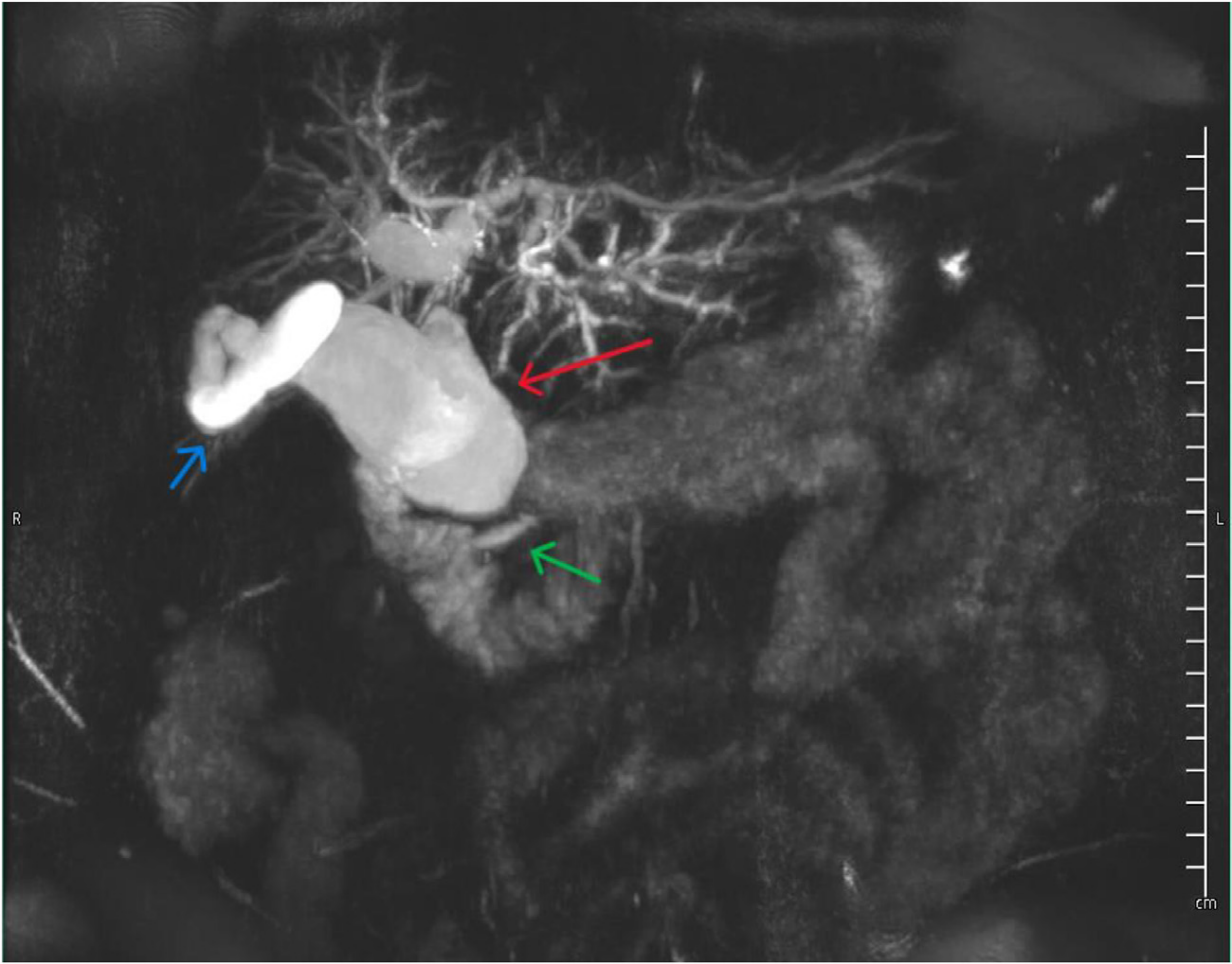
Abdominal MRI/ MRCP coronal Maximum Intensity Projection reconstructed image shows a long common channel (green arrow) suggestive of PBM. Significant dilation of the common bile duct (red arrow) is noted into which the gallbladder and prominent cystic duct drain (blue arrow) consistent with Type 1A choledochal cyst.

**Fig. 3:**
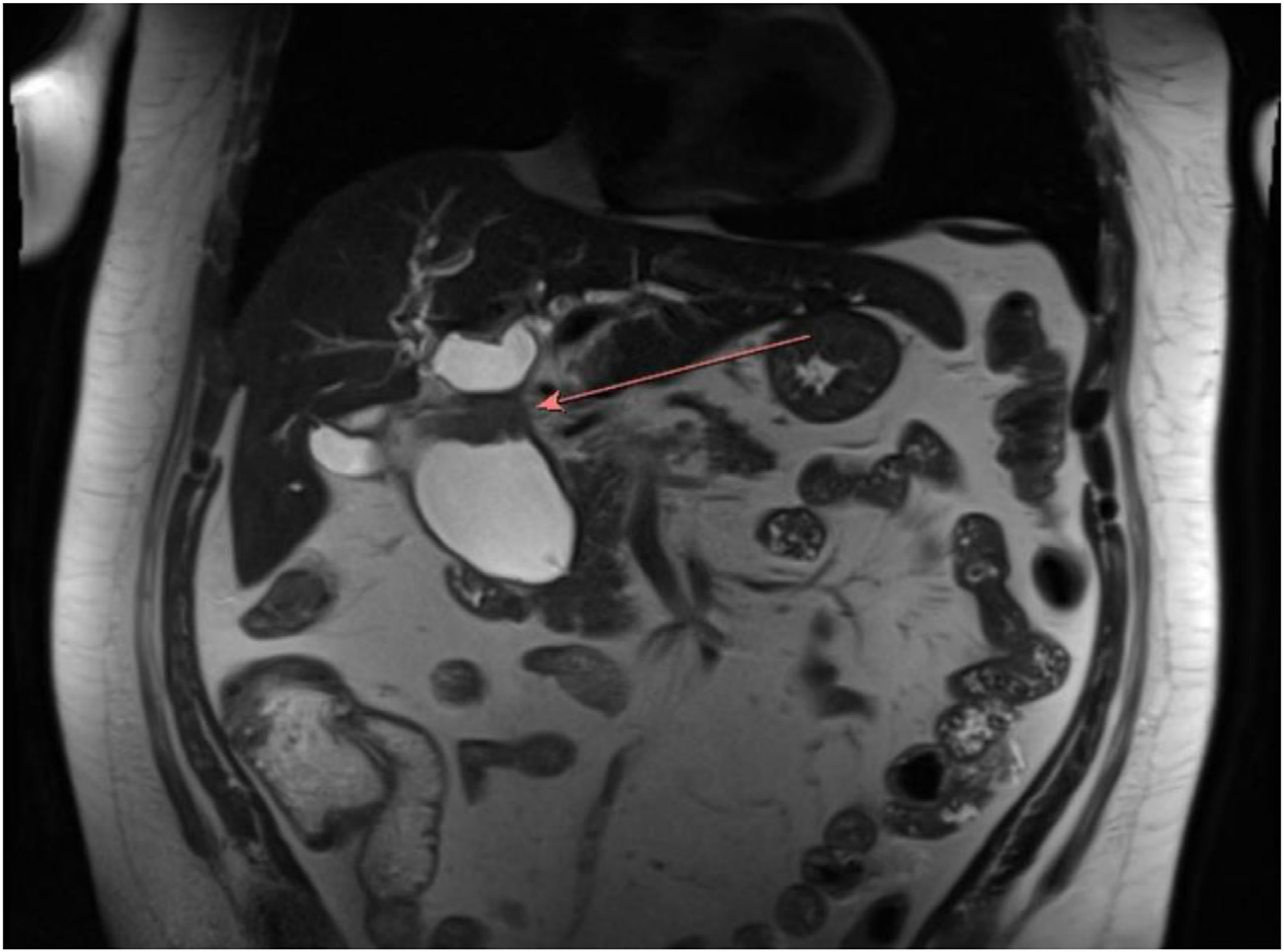
T2 weighted MRI/ MRCP coronal image of the upper abdomen without intravenous contrast shows marked abnormal dilation of the extrahepatic biliary tree suggestive of choledochal cyst. The arrow indicates focal, mass-like thickening of the distal common hepatic duct with associated stricture highly concerning for malignancy. Upstream dilation of the common hepatic duct and intrahepatic biliary tree is noted.

**Fig. 4:**
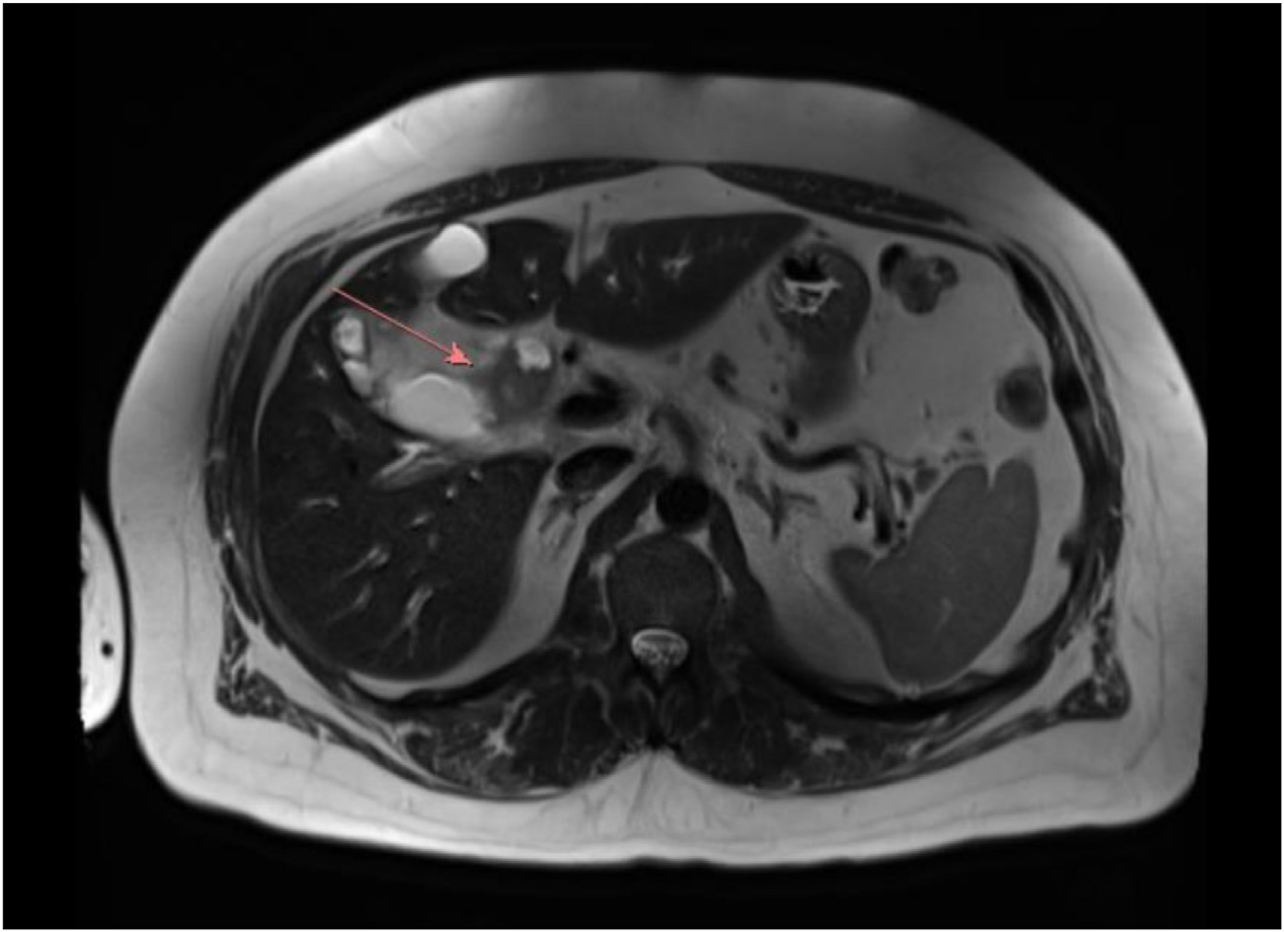
The same T2 weighted MRI/ MRCP with axial image through the upper abdomen again shows focal, mass-like wall thickening of the distal common hepatic duct (red arrow) with upstream dilation of the proximal common hepatic duct.

**Fig. 5:**
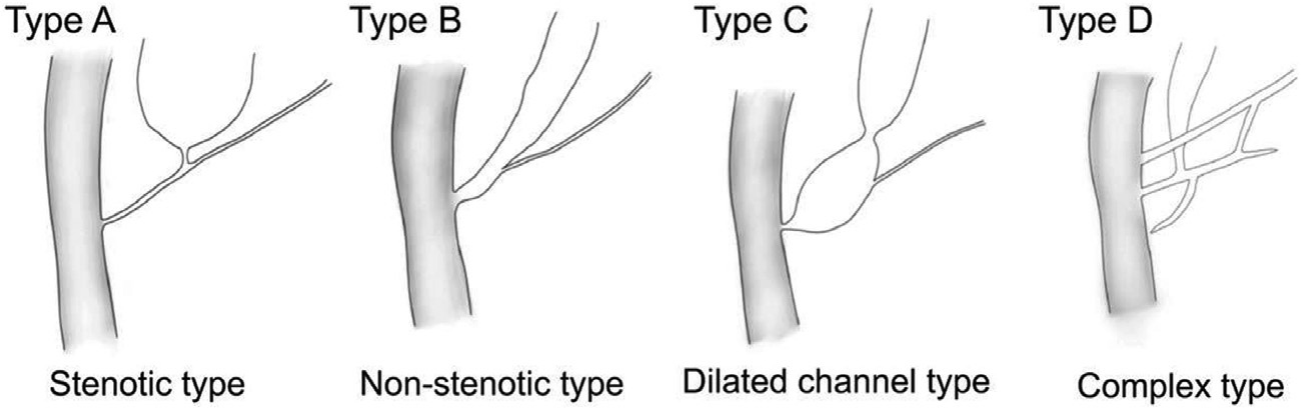
Classification of PBM proposed by the JSPBM. Type A = stenotic type, in which a distal common bile duct with stenosis joins the common channel. Type B = nonstenotic type, in which a distal common bile duct without stenosis joins the common channel. Type C = dilated channel type, in which the common channel is dilated. Type D = complex type, in which the PBJ has formed in a complicated configuration [9].

Given the clinical concern for biliary obstruction, the patient underwent diagnostic work-up including abdominal CT, abdominal MRI with MRCP, and ERCP. The constellation of imaging and endoscopic findings identified anomalous biliary anatomy with pancreaticobiliary maljunction, featuring a long common channel approximately 15 mm in length into which both the pancreatic duct and common bile duct drained. The common bile duct entered this channel through a narrowed distal segment, suggestive of a stricture. Additionally, the common bile duct was severely and diffusely dilated, showing fusiform dilation with a maximum diameter of 50 mm, consistent with a Type 1A choledochal cyst due to the involvement of the cyst duct. Furthermore, the MRI/MRCP revealed a mass-like abnormality in the common hepatic duct, which was highly concerning for extrahepatic cholangiocarcinoma. The diagnosis of extrahepatic cholangiocarcinoma was confirmed with subsequent ERCP and tissue sampling. A PET/CT revealed focal hypermetabolic activity fusing to common bile duct, consistent with biopsy proven malignancy. Otherwise, PET/CT revealed no evidence of distant metastases (Figs. 1, 2, 3, Fig. 4, Fig. 5).

A biliary sphincterotomy was performed, during which a few small stones were successfully drained from the bile duct. The procedure involved dilating the biliary opening at the common channel. Cytology samples were collected from the upper third of the main bile duct for further analysis. Additionally, a plastic stent was placed into the common bile duct for relief of the underlying biliary obstruction. Furthermore, another plastic stent was inserted into the ventral pancreatic duct to minimize the risk of postendoscopic pancreatitis (PEP). Based on the endoscopic and imaging evaluation, the extrahepatic biliary mass was deemed amenable to upfront surgical resection. The patient underwent diagnostic laparoscopy, robotic-assisted bile duct resection, cholecystectomy, portal lymphadenectomy, right hepatic lobectomy (due to right hepatic artery involvement), and Roux-en-Y hepaticojejunostomy.

The surgical specimen revealed an invasive, poorly differentiated adenosquamous carcinoma originating from an intraductal tubular papillary neoplasm with high-grade dysplasia in the upper common bile duct and common hepatic duct. The tumor extended to the radial margin, but the proximal and distal margins were free of malignancy. Perineural invasion was noted, and the right hepatic artery was positive for both invasive carcinoma and perineural invasion. The portal lymphadenectomy showed no involvement of portal or regional lymph nodes.

## Discussion

Pancreaticobiliary maljunction (PBM) is a congenital anomaly where the pancreatic and bile ducts join outside the duodenal wall, resulting in formation of an elongated common channel [1]. In normal physiology, the sphincter of Oddi regulates the junction between the pancreatic and bile ducts. Individuals with PBM lack this regulatory mechanism resulting in reflux of pancreatic juices into the biliary tract. According to Babbitt's theory, activated pancreatic enzymes result in chronic inflammation of biliary tract resulting in choledochal cysts which places patients at risk for eventual development of cholangiocarcinoma [2]. This patient has a rare triad of pancreaticobiliary maljunction, choledochal cyst, and cholangiocarcinoma. Choledochal cysts (CCs) are rare in Western populations, with an occurrence rate of 1 in 100,000 to 150,000 live births. However, in the United States, the incidence is higher, reported at 1 in 13,500 births, and in Australia, at 1 in 15,000 births. In Asian populations, especially in Japan, the incidence of choledochal cysts is significantly higher, estimated at 1 in 1,000 births [3]. Moreover, the incidence of Pancreaticobiliary Maljunction is 100 to 1000 times more frequent in Asians compared to other regions globally [4]. On the other hand, the incidence of PBM is estimated to be about 1 in 100,000 in Western populations [5]. PBM is more common in women than in men, with a female-to-male ratio of 3:1 [6]. Timely detection and surgical intervention are imperative for good prognosis. Diagnosing PBM, choledochal cysts and cholangiocarcinoma relies significantly on imaging and endoscopic findings, with advanced techniques such as multidetector CT and MR cholangiopancreatography playing a crucial role in identifying PBM and its possible complications [4]. Furthermore, the imaging and endoscopic evaluations are critical for surgical planning.

The Japanese Study Group on Pancreaticobiliary Maljunction (JSPBM) has established diagnostic criteria for PBM based on imaging features and anatomical examinations. Diagnosis is confirmed by identifying an abnormally long common channel, an unusual union between the pancreatic and bile ducts, or a PBJ located outside the duodenal wall. There is no set definition of long common channel, but many researchers define it to be more than 10 mm in patients with PBM [7].

Pancreaticobiliary malfunction can be evident on imaging applications such as direct cholangiography, such as ERCP, percutaneous transhepatic cholangiography, or intraoperative cholangiography; MR cholangiopancreatography; or 3D drip infusion CT cholangiography. Diagnosis can be confirmed if PBJ outside the duodenal wall is evident on endoscopic US or on MPR images obtained at multidetector CT [8]. PBM can also be diagnosed anatomically during surgery or autopsy in which PBM lies outside the duodenal wall or that the pancreatic and bile ducts unite abnormally. Supplementary findings that can assist with the diagnosis are elevated levels of pancreatic enzymes, especially amylase, in the bile within the bile duct and gallbladder obtained immediately after the laparotomy [8].

Risk-reducing surgery should be considered in patients with PBM due to potential of developing biliary neoplasms. In patients with PBM with biliary dilatation, most biliary cancers are located in the gallbladder or dilated common bile duct [6]. Therefore, in patients with biliary dilatation, resection of the extrahepatic bile duct along with the gallbladder is considered the standard surgical procedure [10]. Ideally, the hepatic side should be transected at the junction where the right and left hepatic ducts converge, while the pancreatic side should be transected just above the point where the bile and pancreatic ducts meet [11].

Hepaticojejunostomy with Roux-en-Y anastomosis is commonly performed and helps prevent postoperative gastritis caused by bile reflux, unlike hepaticoduodenostomy [12]. Recent reports suggest that partial hepatectomy during the initial surgery should be considered for adult patients with primary biliary malignancy and both extrahepatic and intrahepatic biliary dilatation to reduce the risk of carcinogenesis and cholangitis [13]. However, hepatectomy is invasive, particularly for pediatric patients, and a consensus has not yet been reached [11].

For patients with Pancreaticobiliary Maljunction who do not have biliary dilatation, prophylactic cholecystectomy is recommended, as most biliary cancers originate in the gallbladder [10]. However, it remains uncertain whether preventive resection of the extrahepatic common bile duct should be carried out [10].

Patients with PBM who have undergone risk reducing surgery must adhere to a periodic imaging schedule for the rest of their lives due to the long-term risk of developing biliary cancer arising from the residual bile ducts [10]. Patients with PBM remain at risk for biliary cancer in residual bile ducts, including intrahepatic and intrapancreatic ducts, even after prophylactic resection of the extrahepatic bile duct [14]. In those with biliary dilatation, the prevalence of residual biliary cancer is 2%, with a mean time of 12 years from surgery to diagnosis of biliary cancer [14].

## Conclusion

This case was a rare triad of pancreaticobiliary maljunction, Type 1A choledochal cyst and cholangiocarcinoma in a 45-year-old male who was diagnosed by a combination of advanced imaging studies and endoscopic evaluation. Advanced imaging, such as MRI/MRCP, plays a crucial in diagnosing pancreaticobiliary maljunction and surveillance of its complications such as choledochal cyst formation and cholangiocarcinoma. Understanding the morphological characteristics of PBM is essential for accurate diagnosis, surgical planning, and surveillance.

## Patient consent

Informed consent was obtained via telephone, with a witness present, from the patient's wife, granting permission to publish this case report. A copy of the informed consent can be provided to the Editor-in-Chief of the journal upon request.
